# Fecal Microbiota Transplantation Protects the Intestinal Mucosal Barrier by Reconstructing the Gut Microbiota in a Murine Model of Sepsis

**DOI:** 10.3389/fcimb.2021.736204

**Published:** 2021-09-22

**Authors:** Xiaowei Gai, Huawei Wang, Yaqing Li, Haotian Zhao, Cong He, Zihui Wang, Heling Zhao

**Affiliations:** ^1^ Department of Intensive Care Unit, Hebei General Hospital, Shijiazhuang, China; ^2^ Graduate School of Hebei Medical University, Hebei Medical University, Shijiazhuang, China; ^3^ Department of Intensive Care Unit, Qinhuangdao Jungong Hospital, Qinhuangdao, China; ^4^ Department of Infection, Hebei General Hospital, Shijiazhuang, China; ^5^ Department of Ultrasound, Hebei General Hospital, Shijiazhuang, China

**Keywords:** sepsis, fecal microbiota transplantation, intestinal mucosal barrier, gut microbiota, critical care

## Abstract

The gastrointestinal (GI) tract has long been hypothesized to play an integral role in the pathophysiology of sepsis, and gut microbiota (GM) dysbiosis may be the key factor. Previous studies have shown that the gut flora was significantly altered in critically ill patients. This study aimed to observe what kind of GM dysbiosis is in the early stage of sepsis and whether the application of fecal microbiota transplantation (FMT) can reconstruct the GM of septic mice and restore its protective function on the intestinal mucosal barrier. The study investigated the effect of FMT on gut microbiota, mucosal barrier function, inflammatory response, and survival in a murine model of sepsis established by cecal ligation and puncture (CLP). It is found that FMT can not only reduce morbidity and mortality and restore the abundance and diversity of the gut flora in septic mice, but can also improve the intestinal barrier function by reducing epithelial cell apoptosis, improving the composition of the mucus layer, upregulating the expression of tight junction proteins, and reducing intestinal permeability and the inflammatory response. After FMT, Lachnospiraceae contributed the most to intestinal protection through enhancement of the L-lysine fermentation pathway. FMT offers a microbe-mediated survival advantage in a murine model of sepsis. Therefore, an improved understanding of the connection between microbiota, and systemic illness may yield new therapeutic strategies for patients with sepsis.

## Introduction

Sepsis continues to be the leading cause of mortality in the intensive care unit (Fay et al., 2017; Haak and Wiersinga, 2017). The World Health Organization has recognized sepsis as a global health priority (Fay et al., 2019). Despite significant advancement in our understanding of the pathophysiology of sepsis, treatment of sepsis is still limited to antibiotics, aggressive fluid resuscitation, vasopressor administration, and supportive care, and no targeted therapeutics for sepsis are approved for usage in patients (Ames et al., 2018).

Sepsis is defined as a life-threatening organ dysfunction caused by a dysregulated host response to infection (Ames et al., 2018; Fay et al., 2019; Gong et al., 2019). The syndrome can be induced by a wide variety of microbes by definition and the gastrointestinal tract is the largest pool of bacteria (Fay et al., 2019). It is known that the human gastrointestinal tract contains trillions of bacteria that comprise a complex ecosystem known as the intestinal microbiota that has relevant implications in human health and disease (Bassetti et al., 2020). The symbiotic relationship between microbiota and the host is mutually beneficial (Ekmekciu et al., 2017). The host provides an important habitat and nutrients for the microbiome, and the gut microbiota supports the development of the metabolic system and the maturation of the intestinal immune system by providing beneficial nutrients, for example, by the synthesis of vitamins and short-chain fatty acids (SCFAs) (Shi et al., 2017). Resident microbiota can out-compete pathogens for space, metabolites, and nutrients, and inhibit pathogens by calibrating the host immune response (Bassetti et al., 2020). However, the microbiome is markedly altered in critical illness. Studies have shown that the microbial diversity is diminished within 6 hours of admission to the intensive care unit, and this lack of diversity has been associated with poor outcomes in critically ill patients (Fay et al., 2019).

Considering GM dysbiosis is one of the most important factors that can lead to pathological bacterial translocation and systemic infection, it may be feasible to develop novel therapeutic strategies against gut-derived sepsis by modulating the microbiota (Wang et al., 2019). More than 90% of the commensal organisms may be lost during the early stage of critical illness, making it nearly impossible that a single or several probiotic species would be able to completely replenish the diversity of the GM without intervention. Transfer of healthy donor feces containing thousands of microbial species, termed FMT, facilitates the replenishment of diminished commensal bacteria and may guide the patient’s microbiota toward a healthy state (Wang et al., 2019). Fecal microbiota transplantation has been successfully applied to a series of diseases (Otani and Coopersmith, 2019; Zeng et al., 2019). Even though the evidence is limited to some case reports on the treatment of septic patients, the improved clinical outcomes following FMT are promising (Schmidt et al., 2018; Limketkai et al., 2019; Wang et al., 2019). However, during sepsis, the exact mechanism of action for the use of FMT on the intestine is still unknown (Haussner et al., 2019). Considering the important role of microbiota in sepsis, we wonder whether the use of fecal microbiota transplantation in the early stage of sepsis can inhibit or even reverse the clinical outcome.

## Materials and Methods

### Animals Experiments

All experimental procedures were performed by the Guide for the Care and Use of Laboratory Animals (U.S. National Institutes of Health) and were approved by the Animal Ethics Committee of Hebei Medical University (identification number: 202152). Male C57BL/6 mice, approximately 6–8-weeks old and weighing 20 –25 g, were purchased from Beijing Vital River Laboratory Animal Technology Co., Ltd. (license number: SCXK(京)2016-0006). The animals were housed in a temperature-controlled environment (20°C-23°C and 45%-55% humidity) with a 12 h light-dark cycle. The mice were allowed to acclimate to the housing conditions for one week before the experiments started. The experiment was divided into two parts: an acute experiment and a 7-day mortality observation experiment. All experimental animals were randomly divided into four groups: the sham operation group, the sepsis model group, the fecal microbiota transplantation group, and the normal group (healthy donor mice). The ten normal mice were only used to collect feces and make fecal bacteria liquid. The mice were euthanized at 12, 24, and 48 hours following cecal ligation and puncture for acute studies, respectively (Sham: n=6 per time point; CLP: n= 10/11 per time point; FMT: n=10/11 per time point). Ten mice in each group of the sham group, the CLP group, and the FMT group were used for a 7-day mortality observation experiment. All animals had free access to food and water. All surgery was performed under anesthesia, and every effort was made to minimize suffering.

### Sepsis Model

Sepsis was induced by cecal ligation and puncture as previously described (Rittirsch et al., 2009; Khailova et al., 2013). Mice were anesthetized using an intraperitoneal injection at 50 mg/kg of 2% sodium pentobarbital. To perform the surgery, the cecum was exposed by a 1.5 cm midline incision in the abdomen. The cecum was ligated at 1cm from the cecal tip using a single suture, punctured with a squeeze to extrude a small amount (droplet) of feces from the perforation sites, and returned to the peritoneal cavity. The location of the cecal ligation and the size of the puncture or hole was determined in each mouse. The amount of extruded cecal content was kept the same to ensure the consistency of the model. The laparotomy was closed with silk sutures. Sham controls were subjected to the same procedures, except that there was no ligation or puncture of the cecum. Animals were resuscitated by subcutaneously injecting pre-warmed normal saline (37°C; 5 mL per 100 g body weight). Recovery of these mice was assessed and recorded 2 hours after surgery, and their survival was recorded daily, including diet, fur, bloating, defecation, mobility, abnormal behavior, and response to stimuli. Tissue, blood, and feces samples of all mice were collected when they were euthanized.

### Fecal Microbiota Transplantation

Fresh feces were collected from ten healthy C57BL/6 mice, homogenized in 10 mL of sterile phosphate-buffered saline (PBS), and centrifuged for 30 sec at 800 ×g, 4°C, to pellet the particulate matter. The optical density (OD) value of the supernatant slurry was checked to calculate the concentration of total bacteria (OD = 0.5 represents 10^8^ cells). For each mouse, 1×10^9.8^ bacterial cells (sum of the total bacterial population within 2 g cecal contents) were centrifuged for 5 min at 13,000 ×g, 4°C, and then bacterial pellets were resuspended in 0.2 mL PBS (Li et al., 2015; Li et al., 2017). Mice in the FMT group received a single dose of fecal microbiota just prior to cecal ligation and puncture and were treated for three consecutive days (Khailova et al., 2013). The mice in the CLP group and the Sham group were gavaged with 0.2 mL PBS once a day as a control.

### Serum IL-6, IL-10, and TNF-α Analysis

Enzyme-linked immunosorbent assay was used to determine the concentrations of TNF-α (Lot: RXQJYSXLD4, Elabscience, China), IL-6 (Lot: 449268JEI6, Elabscience, China), and IL-10 (Lot: C8QEY2KAVE, Elabscience, China) in serum according to the manufacturer’s instructions. Serum was collected after centrifuging blood for 10 min at 4°C and 800 ×g and stored at -80°C until the assay was performed.

### Sample Processing for Animal Experiments

After the mice were euthanized, their GI tracts were quickly removed. The colons were gently separated, by cutting at the cecum-colon junction and rectum and divided into two parts. One-half of each colon was immediately frozen in liquid nitrogen and then stored at -80°C until further use. The other part ones were preserved in Carnoy’s fixative (dry methanol: chloroform: glacial acetic acid in the ratio 60:30:10) (Johansson et al., 2011; Desai et al., 2016). The Carnoy’s fixative was made fresh with anhydrous methanol, chloroform, and glacial acetic acid. The colons were fixed in Carnoy’s solution for 3 h followed by transfer to fresh Carnoy’s solution for 2-3 h. The colons were then washed in dry methanol for 2 h, placed in cassettes, and stored in fresh dry methanol at 4°C. Samples were then embedded in paraffin, and cut into sections (5μm thick). Cecal contents from each animal were divided into replicates, and they were all instantly flash-frozen in liquid nitrogen and then stored at -80°C until the use for microbiome evaluation.

### Immunohistochemistry and Immunofluorescence

The colonic sections were mounted onto polylysine-coated slides, deparaffinized, rehydrated, and placed in a 3% citrate buffer to repair antigens. After being pretreated with 3% H2O2 for 30 min, the sections were blocked with goat serum for 20 min. Sections were incubated overnight at 4°C with a rabbit polyclonal to MUC2 blocking antibody (MUC2, ab90007, Abcam Ltd.; Occludin, ab168986, Abcam Ltd; caspase 3, ab44976, Abcam Ltd.). The sections were washed with PBS and incubated with a secondary antibody for 30 min, rewashed, and incubated with peroxidase-conjugated streptavidin for 30 min. DAB developed, hematoxylin counterstained, dehydrated, and mounted. The secondary antibody of MUC 2 was prepared in 0.5% Triton X-100 PBS buffer, and the Alexa Fluor™ 488 donkey anti-rabbit antibody was diluted 1:1000. The specimen was incubated in this solution at 37°C for 1 h. We aspirated the secondary antibody and rinsed the specimen three times in PBS for 5 min each and covered the specimen with a DAPI coverslip.

### Transmission Electron Microscopy (TEM)

For TEM, two 0.5×0.5 cm mini-segments of intestinal tissue from each group were excised and placed in a fixative for TEM at 4°C for 2-4 h. The segments were washed in 0.1 M PBS three times for 15 min each time, postfixed in 1% osmium tetroxide in PBS, dehydrated in a graduated series of ethanol solution, and embedded by baking in an oven at 60°C for 48 h. Samples were cut into 60-80 nm sections and stained with uranyl acetate and lead citrate. The sections were analyzed by electronic microscopy (HT7700 TEM; Hitachi Inc., Tokyo, Japan).

### Western Blot

The snap-frozen tissues were subjected to homogenization in 250μL of lysis buffer as previously described. Samples (30 μg of protein for each condition) were transferred onto PVDF membranes and then incubated with antibodies (Li et al., 2017). The following were used as primary antibodies: caspase 3 (ab323519, Abcam), myeloid differentiation factor 88 (MyD88) (SC74532, Santa Cruz), toll-like receptor 4 (TLR4) (AF7017, Affinity), ZO-1 (AF 5145, Affinity), occludin (DF7504, Affinity), and NF-κB(ab16502, Abcam). Immunoreactive bands were revealed using a 1:10,000 dilution of secondary antibody conjugated to horseradish peroxidase (goat anti-rabbit IgG, BE0101, Bioeasy; goat anti-mice IgG, BE0102, Bioeasy). The blots were re-probed with antibodies against β-actin (EASYBIO) and GAPDH (Bioworld) to ensure equal loading and transfer of proteins. All critical blots and immunoprecipitation experiments were repeated at least three times.

### Real-Time PCR

Total RNA was isolated from colonic tissue using the RNA simple Total RNA Kit (Tiangen Biotech, Beijing, China) as described in the manufacturer’s protocol. The RNA concentrations were quantified at 260 nm, and their purity and integrity were determined using a NanoDrop. Reverse transcription and real-time PCR assays were performed to quantify steady-state messenger RNA levels of TLR4, MyD88, and NF-κB at 48 h following CLP. Complementary DNA was synthesized from 0.1 μg of total RNA. The following cycling protocol was used: denaturation at 95°C (15 min) and 40 cycles of 95°C (10 s), 60°C (32 s). The reporter dye emission (SYBR green) was detected by an automated sequence detector combined with ABI Prism 7500 Real-Time PCR System (Applied Biosystems, Foster City, CA, USA).

### Microbiome Evaluation

Fecal samples were collected at the time mice were euthanized and frozen until DNA extraction samples were sent to the Shanghai Personal Biotechnology Co., Ltd., where bacterial DNA was extracted. PCR amplification of the bacterial 16S rRNA genes V3-V4 region was performed using the forward primer 338F (5’-ACTCCTACGGGAGGCAGCA-3’) and the reverse primer 806R (5’-GGACTACHVGGGTWTCTAAT-3’). The Illumina MiSeq sequencing platform was amplified (Illumina, San Diego, CA, USA) using the NovaSeq-PE250 sequencing strategy. Sequence denoising or OTU clustering was performed according to the QIIME2 dada 2 analysis process or the Vsearch software analysis process. The resulting sequences were clustered with 100% similarity against the GreenGenes to the database (v. 13.8; https://greengenes.secondgenome.com/) (Fay et al., 2019) to assign bacterial operational taxonomic units. Alpha and beta diversity comparisons were performed using QIIME2 and taxonomic summaries were generated with QIIME2. Comparison of sample compositions and identification of statistically significant differences were performed with LEfSe using the correction for independent comparisons. Microbial functions were predicted by using PICRUSt (Phylogenetic investigation of communities by reconstruction of unobserved states) based on high-quality sequences.

### Statistical Analysis

The Kaplan-Meier estimator was used to draw the survival curve of the mice, and the log-rank method was used to compare the survival rates between different groups. The measurement data had a normal distribution, the variance was uniform, and the one-way ANOVA and LSD tests were used for comparison between multiple groups. The Student’s t-test was used to compare the two independent groups, and the measurement result was expressed as the mean ± standard deviation (mean ± sd). The Kruskal-Wallis *H* test was used for data with non-normal distribution and/or uneven variance. The results were expressed in medians (interquartile range). SPSS 21.0, GraphPad Prism 8.0, Photoshop CS5, Image-Pro Plus, and ImageJ were used for data analysis, and *P* < 0.05 was considered statistically significant.

## Results

### Mortality Among Three Groups

**Figure 1:**
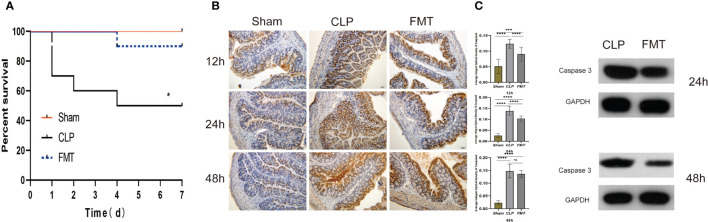
Survival analysis, and caspase 3 expressions among the Sham, CLP, and FMT groups. **(A)** Seven-day mortality observations in the Sham (red), CLP (black), and FMT groups (blue). There were significant differences between the CLP group and the other two groups, *P* < 0.05, but there was no significant difference between the FMT group and the Sham group; **(B)** Immunohistochemistry of the colon [under a digital microscope (20 × 10)]. The average integral optical density of caspase 3 between the Sham, CLP, and FMT groups at 12, 24, and 48 h are shown on the right side. **(C)** Relative expression of caspase 3 compared with GAPDH between the CLP and FMT groups at 24 and 48 h. *P < 0.05, ***P < 0.001, ****P < 0.0001, ns, no statistical difference, respectively.

The survival rates of the Sham group, CLP group, and FMT group were compared 7 days following sepsis modeling. The CLP group had a mortality rate of 30% at 24 h and a 50% survival rate at 7 days. There were no deaths at 24 h in the FMT group, and a 90% survival at 7 days, and no deaths in the Sham group. Compared with the Sham group and the FMT group, the mortality of the CLP group was significantly higher, *P* < 0.05 (**Figure 1A**).

### Apoptosis

The expression of caspase 3 in the CLP group was significantly higher than that of the other two groups at 12 and 24 h, but the average integral optical density (IOD) of caspase 3 in the FMT group was close to that of the CLP group at 48 h (**Figure 1B**). Therefore, we performed a quantitative analysis of caspase 3 protein. The results of the western blot showed that the expression of caspase 3 in the CLP group at 24 and 48 h was higher than that in the FMT group, *P* < 0.001 (**Figure 1C**).

### Mouse Serum Inflammatory Factors TNF-α, IL-6, and IL-10

**Figure 2:**
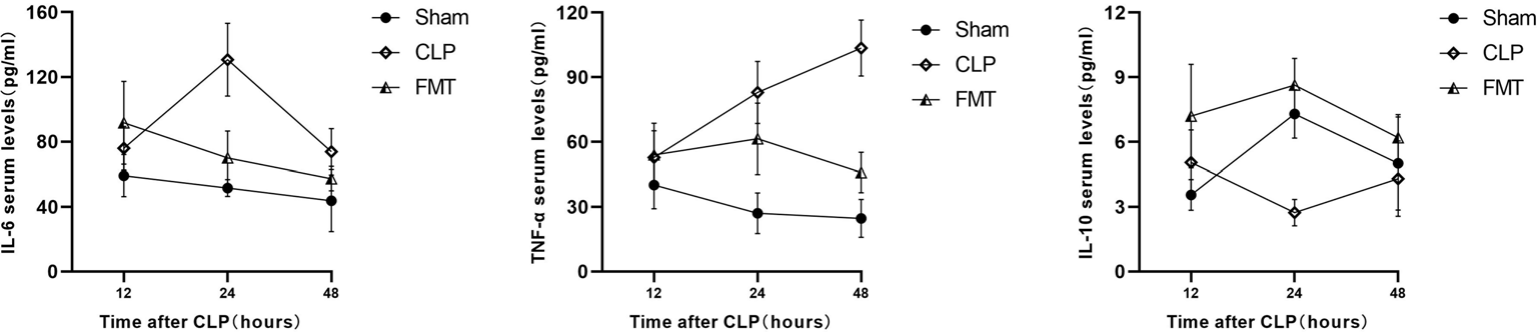
The serum IL-6, TNF-α, and IL-10 among the Sham, CLP, and FMT groups at 12, 24, and 48 h. The concentration of serum IL-6 (pg/mL) and IL-10 (pg/mL) in the FMT group at 12 h after sepsis modeling were significantly higher than in the Sham group (*P* < 0.05), and there was no significant difference in TNF-α levels (pg/mL) among the three groups. The serum IL-6 level in the CLP group was significantly higher than in the Sham group and the FMT group (*P* < 0.001) at 24 h. The TNF-α levels in both the CLP and FMT groups were higher than in the Sham group (*P* < 0.001 and *P* < 0.01, respectively). The IL-10 level in the CLP group was lower than in the Sham group and the FMT group (*P* < 0.001). The serum IL-6 level in the CLP group was higher than in the Sham group at 48 h (*P* < 0.01). The TNF-α level in the CLP group was higher than in the Sham group and FMT group (*P* < 0.001). The TNF-α level in the FMT group was higher than in the Sham group (*P* < 0.05). There was no significant difference among the three groups in the IL-10 level at 48 h.

The expression of IL-6 in the CLP group was the highest at 24 h after modeling, and then decreased, but it was still higher than that in the Sham group at 48 h. Expression of IL-6 in the FMT group was slightly higher than that in the CLP group at 12 h, but there was no significant difference, to the contrary, IL-6 levels were markedly lower than the CLP group at 24 and 48 h. The TNF-α level in the CLP group continued to increase, and it was the highest among the three groups at 48 h, however, the IL-10 level was lowest at 24 h after modeling (**Figure 2**).

### The Thickness of the Mucus Layer (nm) and MUC2 Expression

**Figure 3:**
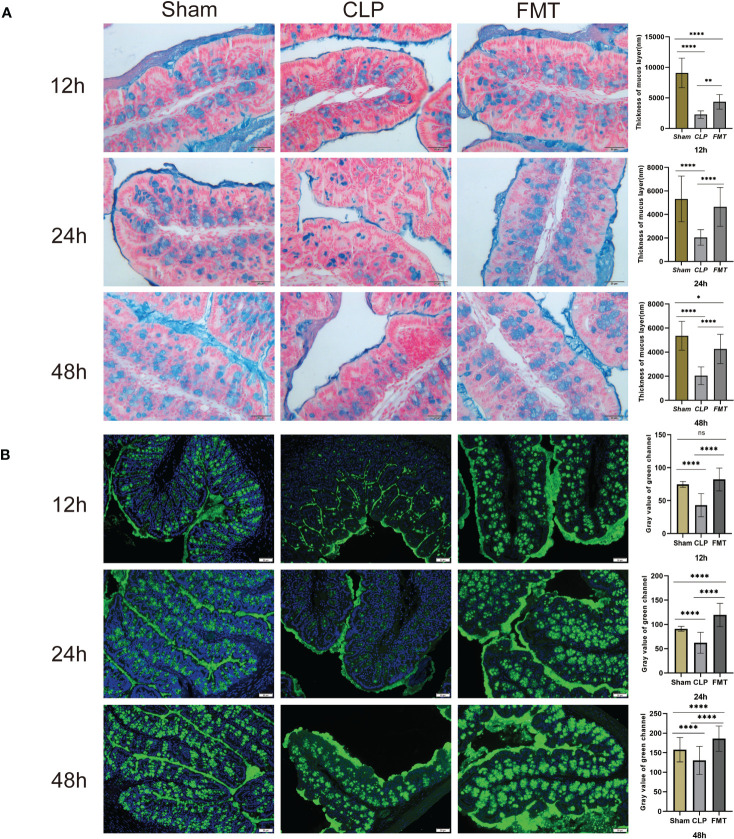
The mucus layer thickness and MUC2 expression in the Sham, CLP, and FMT groups at 12, 24, and 48 h. **(A)** The AB-PAS method was used to measure the thickness of the mucus layer in the Sham, CLP, and FMT groups at 12, 24, and 48 h under a digital microscope (20 × 10). The blue color indicates goblet cell secretion and a mucus layer. The histogram on the right shows the statistical results of the comparison of the three groups. There was no significant difference between the Sham group and the FMT group in mucus layer thickness at 24 h. **(B)** MUC2 expression (green fluorescence) was observed in the three groups at 12, 24, and 48 h. The histogram shows the statistical results of all three groups. There was no significant difference between the Sham group and the FMT group at 12 h. *P < 0.05, **P < 0.01, ****P < 0.0001, ns, no significant difference.

The AB-PAS method was used to detect the colonic mucus layer thickness at 12, 24, and 48 h after sepsis modeling in the three groups. The mucus layer thickness of mice in the CLP group was significantly lower than that of the Sham group during the same timepoint (*P* < 0.0001). The thickness of the mucus layer in the FMT group was significantly greater than that in the CLP group(*P* < 0.01). Compared with the Sham group, the thickness of the mucus layer in the FMT group had no difference at 24 h (*P* = 0.4473) but had a significant difference at 12 and 48 h (*P* < 0.0001, and *P* < 0.05, respectively) (**Figure 3A**). The fluorescence expressions of MUC2 at 12, 24, and 48 h in the three groups were observed by digital microscope and recorded, and the gray value of the green channel was calculated and statistically analyzed. Except that the expression levels of MUC2 in the Sham group and the FMT group were not statistically different at 12 h, there was a significant difference between each pair at 24 and 48 h (*P* < 0.0001). The expression of MUC2 in the CLP group at 12, 24, and 48 h was lower than that of the other two groups (**Figure 3B**).

### Transmission Electron Microscope

**Figure 4:**
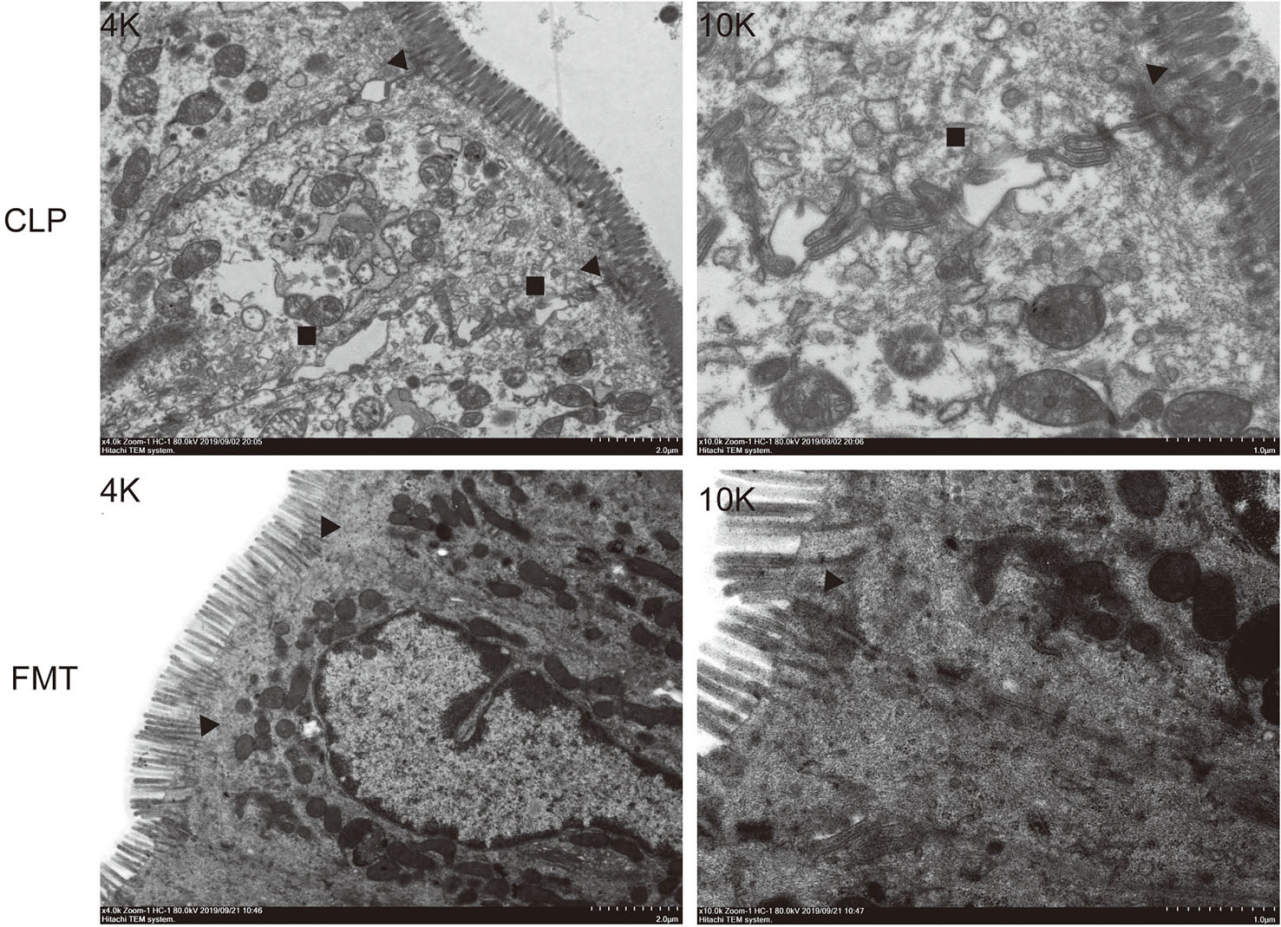
Intestinal epithelial cell junctions. Intestinal epithelial cell junctions in the CLP group and FMT group at 48 h. The images on the left were enlarged 4K, and those on the right were enlarged 10K. ▲ Tight junction and the intermediate junction between intestinal epithelial cells. ■ The space between intestinal epithelial cells.

Intestinal epithelial cells and intracytoplasmic organelles in the CLP group were significantly swollen, and the microvilli were arranged neatly, with uniform thickness and partial shedding. The tight junctions and the structure of the intermediate junctions were blurred, and the gap between junctions was slightly widened in the CLP group. Portions of the desmosomes and tension wires had disappeared. The intercellular space was widened and the mitochondria had swelled. Intestinal epithelial cells and intracytoplasmic organelles in the FMT group were slightly swollen. The microvilli were arranged neatly and uniformly in thickness, and the local area was slightly detached. The tight junctions between epithelial cells and the structure of the intermediate junctions were fuzzy, and the gap was slightly widened in the FMT group. The number of desmosomes was slightly reduced, the surrounding tension filaments were abundant, and the mitochondria were slightly swollen (**Figure 4**).

### Tight Junction Proteins

**Figure 5:**
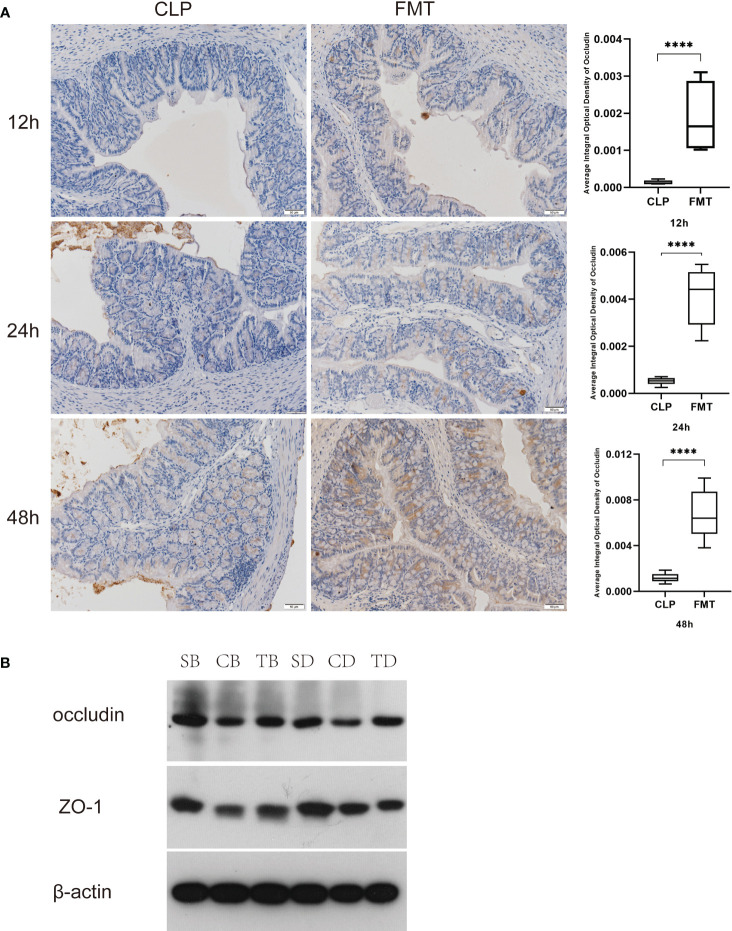
Comparison of tight junction protein expression. **(A)** Comparison of occluding expression in the CLP and FMT groups at 12, 24 add 48 h, respectively, and observed under a digital microscope (20 × 10). Significant results are shown in the box plot on the right, ****P < 0.0001; **(B)** Relative expression of occludin and ZO-1 compared with β-actin expression in the Sham, CLP, and FMT groups at 24 or 48 h. SB, CB, TB, SD, CD, and TD represent the 24 h Sham, CLP, and FMT groups and 48 h Sham, CLP, and FMT groups, respectively.

We compared the average integral OD of occludin between the CLP and the FMT groups at 12, 24, and 48 h. The results showed that occludin expression in the CLP group at 12, 24, and 48 h was significantly lower than expression in the FMT group (**Figure 5A**). We further verified occludin and ZO-1 protein expression by western blot test. The results showed the relative expression of these two proteins in the CLP group was significantly lower than that in the other two groups at 24 or 48 h, (*P* < 0.001). The relative expression of the two proteins was highest in the Sham group, intermediate in the FMT group and lowest in the CLP group (**Figure 5B**).

### TLR4, MyD88, and NF-κB Protein Levels and mRNA Levels

**Figure 6:**
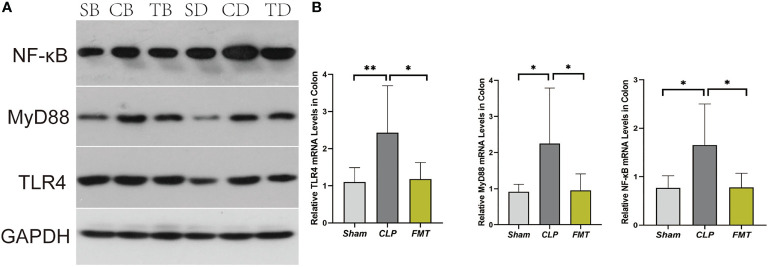
TLR4, MyD88, and NF-κB expression and mRNA levels. **(A)** Expression of TLR4, MyD88, and NF-κB compared to GAPDH protein among Sham, CLP, and FMT groups at 24 and 48 h. SB, CB, TB, SD, CD, and TD represent 24 h Sham, CLP, and FMT groups and 48 h Sham, CLP, and FMT groups, respectively. **(B)** Relative TLR4, MyD88, and NF-κB levels in the colon. *P < 0.05, **P < 0.01.

We analyzed and compared the expression of TLR4, MyD88, and NF-κB relative to GAPDH at 24 and 48 h in the three groups. Except that there was no difference in the expression of TLR4 protein between the Sham and FMT groups, expression levels of the other two proteins in the three groups were significantly different compared with each other. Expression of the three proteins in the CLP group at 24 and 48 h was significantly higher than those in both the Sham and the FMT groups at the same time points (*P* < 0.05) (**Figure 6A**). Coincidently, mRNA expression trends in the three groups were similar to the trends in protein expression. The relative expressions of TLR4, MyD88, and NF-κB, in the CLP group, were significantly higher than those in the Sham and FMT groups. Expression of MyD88, and NF-κB had a significant difference in the CLP group compared with the Sham and FMT groups(*P* < 0.05). There was no difference in the expression of the three indicators between the Sham group and the FMT group (**Figure 6B**).

### 16SrRNA Sequence Analysis

#### Krona Species Composition

**Figure 7:**
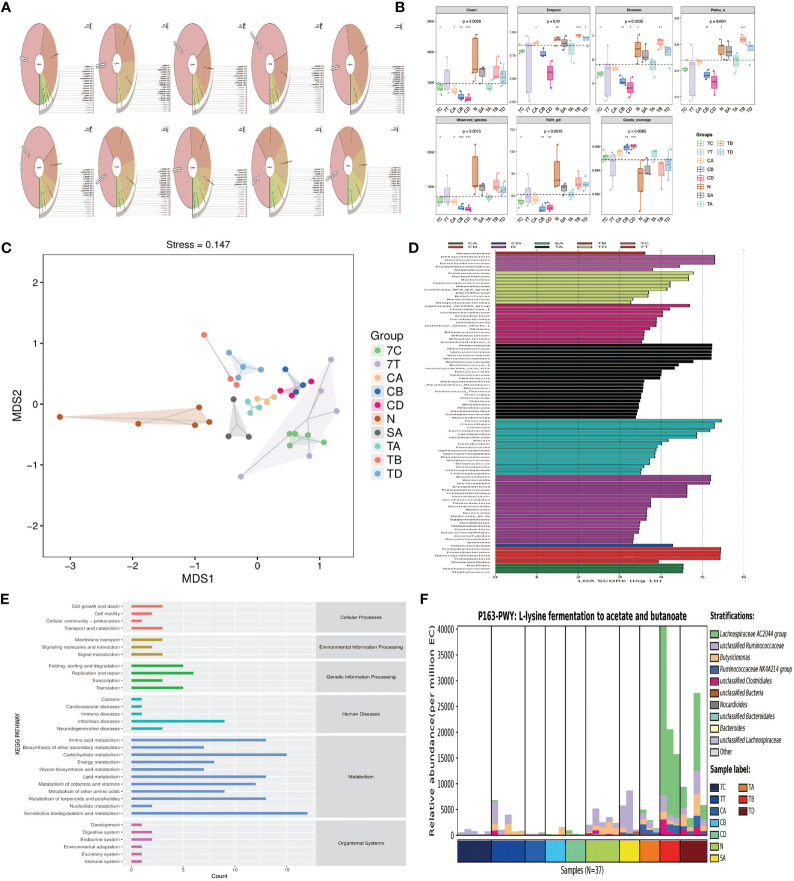
Microbiota analysis. **(A)** Krona species at phylum among the Normal, Sham, CLP, and FMT groups. **(B)** Alpha diversity. *P < 0.05, **P < 0.01 ****P < 0.001. **(C)** NMDS two-dimensional sorting diagram. The closer the distance between the two points in the figure, the smaller the difference between the microbial communities in the two samples. **(D)** LEfSe (LAD Effect Size). A longer bar denotes a more significant difference in the taxon. The color of the bar graph indicates the most abundant sample group corresponding to the taxon. **(E)** Predicted abundance map of KEGG secondary functional pathways. **(F)** Species composition map of differential MetaCys metabolic pathways. The abscissa shows different groups. The order of the samples in the groups was sorted according to the similarity of the data; the ordinate was the relative abundance of the metabolic pathways, and the species different levels of contribution to the metabolic pathways were displayed in different colors at different levels. (In order to show the results better, we used abbreviations to represent each group: N = normal mice, 7C = CLP group mortality observation, 7T = FMT group mortality observation, SA = Sham group 12 h; CA, CB, and CD = CLP group at 12, 24, and 48 h time points, respectively, and TA, TB, and TD = FMT group at 12, 24, and 48 h time points, respectively).

Firmicutes and Bacteroidetes were the main bacteria, accounting for 55% and 33% of the abundance found in the normal mice, respectively. The Sham group was dominated by Firmicutes, at 66%. Proteobacteria was the dominant bacteria in the CLP group. The relative abundance of Proteobacteria observed in the mortality observation of the CLP groups following sepsis modeling at 12 h, 24 h, and 7-day were 48%, 66%, and 42%, respectively. The relative abundance of unclassified bacteria in the CLP group at 48 h and the 7-day mortality observation in the FMT group was 70% and 41%. The FMT group at 24 and 48 h were dominated by Firmicutes and Bacteroidetes, accounting for 48%, 27%, and 37%, 26%, and the composition closely resembled the composition found in mice. The main bacteria in the FMT group at 12 h were Proteobacteria and Verrucomicrobiae, with a relative abundance of 33% and 39%, respectively. Verrucomicrobiae accounted for 22% in the CLP group at 24 h (**Figure 7A**).

### Alpha Diversity Analysis

The 12 h Sham group had a similar fecal richness and diversity compared with normal mice. The fecal richness and diversity were significantly lower in the CLP group at 12, 24, and 48 h and 7-day mortality than those of normal mice. We observed that the fecal richness and diversity were lowest at 48 h in the CLP group, but there was a slight recovery in the 7-day still alive mice in the CLP group, a significant difference compared with the normal mice. The richness and diversity of the flora in the FMT group were higher than that observed in the CLP group at all time points, and there was more fecal diversity than the normal mice at 24 h in the FMT group. There was no significant difference in fecal richness and diversity in the 7-day mortality FMT mice compared with the CLP group at the same timepoint (**Figure 7B**).

### Beta Diversity Analysis

The dimensionality reduction of multi-dimensional microbial data was performed through NMDS analysis, and the main trends of data changes were displayed through the distribution of samples on a continuous sorting axis. The data were also classified by cluster analysis. In the NMDS analysis, the clusters within the group were well and the difference between the groups was large (**Figure 7C**).

### LefSe (LDA Effect Size) Analysis

LEfSe analysis can directly perform simultaneous difference analysis on all classification levels, and at the same time, it emphasizes finding robust differences between groups, that is, marker species. The main species found in normal mice were Firmicutes and Bacteroides. The species with high levels in the Sham group were Firmicutes and Lactobacillus, while the differential species in the CLP group were Bacillales and Staphylococcaceae at 12 h, Enterobacteriales and Proteobacteria at 24 h, Planococcaceae at 48 h, and Deltaproteobacteria, Desulfovibrionales, and Erysipelatoclostridium at 7-day mortality. The FMT group was modeled with high levels of bacteria, including Verrucomicrobiae, Akkermansia, and Ruminococcus at 12 h, Lachnospiraceae group, Bifidobacteriales, Actinobacteria at 24 h, and Burkholderiaceae, Bacteroides, and Butyricimonas at 48 h (**Figure 7D**).

### Functional Analysis

The core of the KEGG database is a biological metabolic pathway analysis database, in which metabolic pathways are classified into six categories, including metabolism, genetic information processing, environmental information processing, cellular processes, organismal systems, and human diseases. We found that infectious diseases had the highest relative abundance among human diseases in the KEGG secondary functional pathway (**Figure 7E**).

### Metabolite Analysis

We further analyzed the species composition of the differential pathways, and found that the Lachnospiraceae contributed the most to L-lysine fermentation to acetate and butanoate (**Figure 7F**).

## Discussion

The commensal microbiome has been shown to play a key role in intestinal immunity because microbes regulate the maturation of the mucosal immune system, support local mucosal immunity, regulate cell growth, and maintain the epithelial barrier function (Haussner et al., 2019). Sepsis alters the composition of the flora and disrupts the balance between the host and the gut (Schmidt et al., 2018; Liu et al., 2019). Striking abnormalities have been reported in the intestinal microbiota of critically ill patients with sepsis, with a wide inter-individual variation and a low bacterial diversity (Avila et al., 2020; Bassetti et al., 2020). In this context, FMT is an effective strategy for adjusting the dysbiosis and restoring the normal gut microflora in patients with sepsis (Avila et al., 2020).

Fecal microbiota transplantation refers to the transplantation of functional bacteria from the feces of healthy donors into the patient’s GI tract to restore the intestinal micro ecological balance and subsequently to treat diseases related to microbial imbalances (Wang et al., 2019; Zeng et al., 2019). This study was an attempt to explore whether FMT can maintain the integrity of the intestinal flora and protect the intestinal barrier function in mice with sepsis. We found that there was a flora imbalance 12-h after the modeling of sepsis. Proteobacteria had an absolute advantage, but Firmicutes and Bacteroidetes decreased, which was consistent with previous studies (Otani and Coopersmith, 2019). The relative abundance of Firmicutes and Bacteroidetes recovered slightly over time, but the amount of Proteobacteria in the intestinal flora of the 7-day mortality CLP group still accounted for 42%, which was significantly higher than that of normal mice. The analysis of the bacterial flora in the FMT group revealed that Firmicutes and Bacteroidetes were the most prevalent and the relative abundance of Proteobacteria was low, meaning that the bacterial composition is similar to that of normal mice. Besides, the Alphaproteobacterial load increased and the Betaproteobacterial load decreased in the FMT group. All of these observations indicate that the intestinal flora of septic mice had been replenished after FMT. It is believed that FMT can maintain the intestinal bacterial balance by increasing the diversity of the flora and restoring and protecting intestinal flora from external interference (Jeon et al., 2018). However, in the observation of the 7-day mortality CLP group, the richness and the diversity of bacterial flora were both lower than those of the FMT group at 24 -h and 48 -h. As aforementioned, the dose and the number of infusions of FMT may have contributed to this result (Jeon et al., 2018). This experiment confirmed that the 7-day mortality rate of septic mice was significantly higher than that of the FMT group. Early application of FMT can effectively reduce the mortality of septic mice, most likely due to the reconstruction of intestinal flora.

The GM supports the development of the metabolic system and the maturation of the intestinal immune system by providing beneficial nutrients, such as synthesizing vitamins and short-chain fatty acids (SCFAs) (Shi et al., 2017). Butyrate, especially, can promote the release of the mucoprotein so as to maintain the mucus barrier (Johansson and Hansson, 2016; Wang et al., 2019). Studies have shown that SCFAs have anti-inflammatory and immunoregulatory activities and may reduce butyrate-producing bacteria such as Ruminococcaceae, Faecalibacterium, and Roseburia (Cammarota et al., 2015). Therefore, we compared the relative abundance of Ruminococcaceae in each group, and the results suggest that fecal bacteria significantly reduced in the sepsis model group, while in the FMT group, the bacterial counts were similar to or even slightly higher than those found in the normal mice. (In our study, the relative abundance of the normal mice was 0.07%, 0.0025% in the CLP group, and 0.067% in the FMT group in 48-h). It appears that Ruminococcaceae plays a role in the inflammatory response, and fecal microbiota transplantation can effectively improve the intestinal bacteria composition, thereby improving the inflammatory state. We found through the functional prediction that the relative abundance of intestinal flora was significantly different in infectious diseases. Further analysis of the species composition of the different pathways revealed that Lachnospiraceae contributed the most to L-lysine fermentation to acetate and butanoate, consistent with previous studies (Shen et al., 2018). This indicated that Lachnospiraceae may be the key bacteria for the effectiveness of FMT.

Tight junctions play an important role in maintaining the integrity of the mucosal epithelium (Zihni et al., 2016; Xu et al., 2018), and it is generally defined as a life-threatening organ failure in the setting of critical illness (Guttman and Finlay, 2009; Vermette et al., 2018). Critical illness induces hyper-permeability of the gut barrier which begins as early as 1 hour after the onset of sepsis and lasts at least 48 hours (Yoseph et al., 2016; Otani and Coopersmith, 2019). We compared transmission electron microscopy results in 48-h between the CLP and the FMT groups. The TJs of the septic mice were blurred more than those of the FMT mice. The cell gap was significantly wider, and both cells and organelles were swollen. We suppose that the mice had not only intestinal barrier dysfunction but also cellular dysfunction. The main functions of occludin are regulating and sealing the TJs (Shawki and McCole, 2017; Wang et al., 2017). That ZO-1 outperformed other TJ markers may reflect the concomitant organ epithelial injury which occurs in MODS (Vermette et al., 2018). Therefore, we observed the expression of the above two proteins in 24-h and 48-h in the three groups, and the results showed that occludin and ZO-1 in the CLP group were significantly lower than those in the Sham group and the FMT group. Levels of the two indicators in the FMT group were higher than those in the CLP group, or they were similar to those in the Sham group.

Research has shown that the thickness of the mucus layer is dependent on commensal bacteria (Otani and Coopersmith, 2019). We performed blinded thickness measurements of the colonic mucus layer in each mouse using Alcian blue-stained sections. We further validated the thickness of the mucus layer by immunofluorescence staining of the MUC2 mucins using a-MUC2 antibody (Desai et al., 2016) and observed that the thickness of the mucus layer in septic mice significantly reduced, and the thickness in the FMT group was significantly increased. This change was consistent with the changes in the flora of the two groups.

TLR4 is the best-characterized pathogen-recognition receptor. Its downstream effects are varied, and the TLR/MyD88/p38 MAPK/NF-κB pathway is popularly believed to play a critical role in the inflammatory response (Piton and Capellier, 2016; Tian et al., 2016). In this study, the TLR4/MyD88/NF-κB pathway in septic mice increased significantly at both the protein level and the gene level, and the inflammatory factors TNF-α and IL-6 increased at different levels in 24-h and 48-h after modeling, while IL-10 decreased in 24-h and 48-h after modeling. FMT treatment decreased the inflammatory response. This is consistent with the pathological score of bowel injury in the three groups. It was observed that the amount of apoptosis protein caspase 3 was different between the CLP group and the FMT group, and the amount of apoptosis in the CLP group was significantly higher than that of the FMT group.

This study has certain limitations: First, we selected the C57BL/6 mouse as our animal model. It is of common knowledge that the human gut microbiota varies greatly, and it is affected by many factors, including host-intrinsic, host-extrinsic, and environmental. Precise microbiome modulation, therefore, is still in its infancy (Schmidt et al., 2018). Second, FMT has been shown to be highly efficient in the treatment of recurrent *C. difficile* infection (Jeon et al., 2018; Schmidt et al., 2018), but its application in sepsis has only been rarely reported. FMT is often preceded by preparatory antibiotic treatment in clinical practice, which makes it difficult to disentangle its effects (Schmidt et al., 2018). While antimicrobials are one of the fundamental and often life-saving modalities in septic patients, they can also pave the way for subsequent harm because of the resulting damage to the gut microbiome (Bhalodi et al., 2019). It has been reported that the association between antibiotic exposure and subsequent sepsis is related to microbiome depletion, rather than the severity of illness (Haak et al., 2018). Therefore, our initial experiment did not involve the use of antibiotics, but its combined application with FMT was included in subsequent studies. Third, it has been reported that the biological activity of the fresh fecal microbiota liquid is not affected by two hours of storage on ice (Hamilton et al., 2012). In our experiment, the fresh fecal bacteria liquid was kept on ice and was transplanted within 1 hour. It is speculated that its biological activity is not affected, but this needs to be confirmed in further research. Fourth, We did not attempt to discern the complete reconstruction time of intestinal flora after initiating FMT, nor did we compare the frequency and the number of transplantation, but it appears that early application of FMT has a protective effect on the intestinal function of septic mice. Finally, in view of the limited predictive function of PICRUSt, we will further use the full RNA transcriptome from both stool and mucosa-adherent microbiota for verification.

## Conclusion

GM imbalance exists early in sepsis. Fecal microbiota transplantation can not only improve morbidity and effectively reduce mortality in septic mice, but can also effectively reduce epithelial cell apoptosis, improve the composition of the mucus layer, upregulate the expression of TJ proteins, and reduce intestinal permeability and the inflammatory response, thus protecting the intestinal barrier function. In our study, after FMT, the abundance and diversity of the gut flora were restored, and the microbial characteristics of the donors changed. Lachnospiraceae contributes the most to intestinal protection through enhancement of the L-lysine fermentation pathway, resulting in the production of acetate and butanoate, and maybe the key bacteria in short-chain fatty acid metabolism that promotes the success of fecal microbiota transplantation.

## Data Availability Statement

The datasets presented in this study can be found in online repositories. The names of the repository/repositories and accessionnumber(s) can be found below: NCBI repository, accession number SRP336491: PRJNA762235.

## Ethics Statement

The animal study was reviewed and approved by The Animal Ethics Committee of Hebei Medical University.

## Author Contributions

XG, HW, and HLZ conceived and designed the experiments, participated in its design and coordination, and helped to draft and revise the manuscript. XG, YL, HTZ, CH, and ZW performed the mice studies and analyzed the data. All authors contributed to the article and approved the submitted version.

## Conflict of Interest

The authors declare that the research was conducted in the absence of any commercial or financial relationships that could be construed as a potential conflict of interest.

## Publisher’s Note

All claims expressed in this article are solely those of the authors and do not necessarily represent those of their affiliated organizations, or those of the publisher, the editors and the reviewers. Any product that may be evaluated in this article, or claim that may be made by its manufacturer, is not guaranteed or endorsed by the publisher.

## References

[B1] Ames S. G. Davis B. S. Angus D. C. Carcillo J. A. Kahn J. M. (2018). Hospital Variation in Risk-Adjusted Pediatric Sepsis Mortality. Pediatr. Crit. Care Med. 19 (5), 390–396. doi: 10.1097/PCC.0000000000001502 29461429 PMC5935525

[B2] Avila P. R. M. Michels M. Vuolo F. Bilesimo R. Burger H. Milioli M. V. M.. (2020). Protective Effects of Fecal Microbiota Transplantation in Sepsis are Independent of the Modulation of the Intestinal Flora. Nutrition 73, 110727. doi: 10.1016/j.nut.2020.110727 32179403

[B3] Bassetti M. Bandera A. Gori A. (2020). Therapeutic Potential of the Gut Microbiota in the Management of Sepsis. Crit. Care 24 (1), 105. doi: 10.1186/s13054-020-2780-3 32204720 PMC7092471

[B4] Bhalodi A. A. van Engelen T. S. R. Virk H. S. Wiersinga W. J. (2019). Impact of Antimicrobial Therapy on the Gut Microbiome. J. Antimicrob. Chemother. 74 (Suppl 1), i6–i15. doi: 10.1093/jac/dky530 30690540 PMC6382031

[B5] Cammarota G. Ianiro G. Cianci R. Bibbo S. Gasbarrini A. Curro D. (2015). The Involvement of Gut Microbiota in Inflammatory Bowel Disease Pathogenesis: Potential for Therapy. Pharmacol. Ther. 149, 191–212. doi: 10.1016/j.pharmthera.2014.12.006 25561343

[B6] Desai M. S. Seekatz A. M. Koropatkin N. M. Kamada N. Hickey C. A. Wolter M.. (2016). A Dietary Fiber-Deprived Gut Microbiota Degrades the Colonic Mucus Barrier and Enhances Pathogen Susceptibility. Cell 167 (5), 1339–1353.e1321. doi: 10.1016/j.cell.2016.10.043 27863247 PMC5131798

[B7] Ekmekciu I. von Klitzing E. Fiebiger U. Escher U. Neumann C. Bacher P.. (2017). Immune Responses to Broad-Spectrum Antibiotic Treatment and Fecal Microbiota Transplantation in Mice. Front. Immunol. 8, 397. doi: 10.3389/fimmu.2017.00397 28469619 PMC5395657

[B8] Fay K. T. Ford M. L. Coopersmith C. M. (2017). The Intestinal Microenvironment in Sepsis. Biochim. Biophys. Acta Mol. Basis Dis. 1863 (10 Pt B), 2574–2583. doi: 10.1016/j.bbadis.2017.03.005 28286161 PMC5589488

[B9] Fay K. T. Klingensmith N. J. Chen C.-W. Zhang W. Sun Y. Morrow K. N.. (2019). The Gut Microbiome Alters Immunophenotype and Survival From Sepsis. FASEB J. 33 (10), 11258–11269. doi: 10.1096/fj.201802188R 31306584 PMC6766641

[B10] Gai X. Wang H. Li Y. Zhao H. He C. Wang Z. Fecal Microbiota Transplantation Reconstructs the Gut Microbiota of Septic Mice and Protects the Intestinal Mucosal Barrier. bioRxiv (2020) 2020.2006.2022.164541.

[B11] Gong S. Yan Z. Liu Z. Niu M. Fang H. Li N.. (2019). *Et Al*: Intestinal Microbiota Mediates the Susceptibility to Polymicrobial Sepsis-Induced Liver Injury by Granisetron Generation in Mice. Hepatology 69 (4), 1751–1767. doi: 10.1002/hep.30361 30506577

[B12] Guttman J. A. Finlay B. B. (2009). Tight Junctions as Targets of Infectious Agents. Biochim. Biophys. Acta 1788 (4), 832–841. doi: 10.1016/j.bbamem.2008.10.028 19059200

[B13] Haak B. W. Prescott H. C. Wiersinga W. J. (2018). Therapeutic Potential of the Gut Microbiota in the Prevention and Treatment of Sepsis. Front. Immunol. 9:2042. doi: 10.3389/fimmu.2018.02042 30250472 PMC6139316

[B14] Haak B. W. Wiersinga W. J. (2017). The Role of the Gut Microbiota in Sepsis. Lancet Gastroenterol. Hepatol. 2 (2), 135–143. doi: 10.1016/s2468-1253(16)30119-4 28403983

[B15] Hamilton M. J. Weingarden A. R. Sadowsky M. J. Khoruts A. (2012). Standardized Frozen Preparation for Transplantation of Fecal Microbiota for Recurrent Clostridium Difficile Infection. Am. J. Gastroenterol. 107 (5), 761–767. doi: 10.1038/ajg.2011.482 22290405

[B16] Haussner F. Chakraborty S. Halbgebauer R. Huber-Lang M. (2019). Challenge to the Intestinal Mucosa During Sepsis. Front. Immunol. 10, 891. doi: 10.3389/fimmu.2019.00891 31114571 PMC6502990

[B17] Jeon S. R. Chai J. Kim C. Lee C. H. (2018). Current Evidence for the Management of Inflammatory Bowel Diseases Using Fecal Microbiota Transplantation. Curr. Infect. Dis. Rep. 20 (8), 21. doi: 10.1007/s11908-018-0627-8 29804272

[B18] Johansson M. E. Hansson G. C. (2016). Immunological Aspects of Intestinal Mucus and Mucins. Nat. Rev. Immunol. 16 (10), 639–649. doi: 10.1038/nri.2016.88 27498766 PMC6435297

[B19] Johansson M. E. Larsson J. M. Hansson G. C. (2011). The Two Mucus Layers of Colon are Organized by the MUC2 Mucin, Whereas the Outer Layer is a Legislator of Host-Microbial Interactions. Proc. Natl. Acad. Sci. U.S.A. 108 Suppl 1, 4659–4665. doi: 10.1073/pnas.1006451107 20615996 PMC3063600

[B20] Khailova L. Frank D. N. Dominguez J. A. Wischmeyer P. E. (2013). Probiotic Administration Reduces Mortality and Improves Intestinal Epithelial Homeostasis in Experimental Sepsis. Anesthesiology 119 (1), 166–177. doi: 10.1097/ALN.0b013e318291c2fc 23571641 PMC5545110

[B21] Li M. Liang P. Li Z. Wang Y. Zhang G. Gao H.. (2015). Fecal Microbiota Transplantation and Bacterial Consortium Transplantation Have Comparable Effects on the Re-Establishment of Mucosal Barrier Function in Mice With Intestinal Dysbiosis. Front. Microbiol. 6, 692. doi: 10.3389/fmicb.2015.00692 26217323 PMC4493656

[B22] Li X. Li X. Shang Q. Gao Z. Hao F. Guo H.. (2017). Fecal Microbiota Transplantation (FMT) Could Reverse the Severity of Experimental Necrotizing Enterocolitis (NEC) *via* Oxidative Stress Modulation. Free Radic. Biol. Med. 108, 32–43. doi: 10.1016/j.freeradbiomed.2017.03.011 28323128

[B23] Limketkai B. N. Hendler S. Ting P. S. Parian A. M. (2019). Fecal Microbiota Transplantation for the Critically Ill Patient. Nutr. Clin. Pract. 34 (1), 73–79. doi: 10.1002/ncp.10228 30561131

[B24] Liu Z. Li N. Fang H. Chen X. Guo Y. Gong S.. (2019). Enteric Dysbiosis is Associated With Sepsis in Patients. FASEB J. 33 (11), 12299–12310. doi: 10.1096/fj.201900398RR 31465241 PMC6902702

[B25] Otani S. Coopersmith C. M. (2019). Gut Integrity in Critical Illness. J. Intensive Care 7, 17. doi: 10.1186/s40560-019-0372-6 30923621 PMC6425574

[B26] Piton G. Capellier G. (2016). Biomarkers of Gut Barrier Failure in the ICU. Curr. Opin. Crit. Care 22 (2), 152–160. doi: 10.1097/MCC.0000000000000283 26808138

[B27] Rittirsch D. Huber-Lang M. S. Flierl M. A. Ward P. A. (2009). Immunodesign of Experimental Sepsis by Cecal Ligation and Puncture. Nat. Protoc. 4 (1), 31–36. doi: 10.1038/nprot.2008.214 19131954 PMC2754226

[B28] Schmidt T. S. B. Raes J. Bork P. (2018). The Human Gut Microbiome: From Association to Modulation. Cell 172 (6), 1198–1215. doi: 10.1016/j.cell.2018.02.044 29522742

[B29] Shawki A. McCole D. F. (2017). Mechanisms of Intestinal Epithelial Barrier Dysfunction by Adherent-Invasive Escherichia Coli. Cell Mol. Gastroenterol. Hepatol. 3 (1), 41–50. doi: 10.1016/j.jcmgh.2016.10.004 28174756 PMC5247418

[B30] Shen Z. H. Zhu C. X. Quan Y. S. Yang Z. Y. Wu S. Luo W. W.. (2018). Relationship Between Intestinal Microbiota and Ulcerative Colitis: Mechanisms and Clinical Application of Probiotics and Fecal Microbiota Transplantation. World J. Gastroenterol. 24 (1), 5–14. doi: 10.3748/wjg.v24.i1.5 29358877 PMC5757125

[B31] Shi N. Li N. Duan X. Niu H. (2017). Interaction Between the Gut Microbiome and Mucosal Immune System. Mil Med. Res. 4, 14. doi: 10.1186/s40779-017-0122-9 28465831 PMC5408367

[B32] Tian Z. Liu J. Liao M. Li W. Zou J. Han X.. (2016). Beneficial Effects of Fecal Microbiota Transplantation on Ulcerative Colitis in Mice. Dig Dis. Sci. 61 (8), 2262–2271. doi: 10.1007/s10620-016-4060-2 26846120

[B33] Vermette D. Hu P. Canarie M. F. Funaro M. Glover J. Pierce R. W. (2018). Tight Junction Structure, Function, and Assessment in the Critically Ill: A Systematic Review. Intensive Care Med. Exp. 6 (1), 37. doi: 10.1186/s40635-018-0203-4 30259344 PMC6158145

[B34] Wang L. Cui Y. L. Zhang Z. Lin Z. F. Chen D. C. (2017). Rhubarb Monomers Protect Intestinal Mucosal Barrier in Sepsis *via* Junction Proteins. Chin. Med. J. (Engl) 130 (10), 1218–1225. doi: 10.4103/0366-6999.205855 28485323 PMC5443029

[B35] Wang G. Huang S. Wang Y. Cai S. Yu H. Liu H.. (2019). Bridging Intestinal Immunity and Gut Microbiota by Metabolites. Cell Mol. Life Sci. 76 (20), 3917–3937. doi: 10.1007/s00018-019-03190-6 31250035 PMC6785585

[B36] Wang C. Li Q. Ren J. (2019). Microbiota-Immune Interaction in the Pathogenesis of Gut-Derived Infection. Front. Immunol. 10:1873. doi: 10.3389/fimmu.2019.01873 31456801 PMC6698791

[B37] Xu J. Liu Z. Zhan W. Jiang R. Yang C. Zhan H.. (2018). Recombinant TsP53 Modulates Intestinal Epithelial Barrier Integrity *via* Upregulation of ZO1 in LPSinduced Septic Mice. Mol. Med. Rep. 17 (1), 1212–1218. doi: 10.3892/mmr.2017.7946 29115466

[B38] Yoseph B. P. Klingensmith N. J. Liang Z. Breed E. R. Burd E. M. Mittal R.. (2016). Mechanisms of Intestinal Barrier Dysfunction in Sepsis. Shock 46 (1), 52–59. doi: 10.1097/SHK.0000000000000565 27299587 PMC4910519

[B39] Zeng W. Shen J. Bo T. Peng L. Xu H. Nasser M. I.. (2019). Cutting Edge: Probiotics and Fecal Microbiota Transplantation in Immunomodulation. J. Immunol. Res. 2019, 1603758. doi: 10.1155/2019/1603758 31143780 PMC6501133

[B40] Zihni C. Mills C. Matter K. Balda M. S. (2016). Tight Junctions: From Simple Barriers to Multifunctional Molecular Gates. Nat. Rev. Mol. Cell Biol. 17 (9), 564–580. doi: 10.1038/nrm.2016.80 27353478

