# Supplementary material for: Fecal Microbiota Transplantation Protects the Intestinal Mucosal Barrier by Reconstructing the Gut Microbiota in a Murine Model of Sepsis
Source: Front Cell Infect Microbiol. 2021 Sep 22;11:736204. doi: 10.3389/fcimb.2021.736204 (PMC8493958; doi:10.3389/fcimb.2021.736204)
Supplement: Supplementary file 1 [file DataSheet_1.doc]

Supplementary figures :


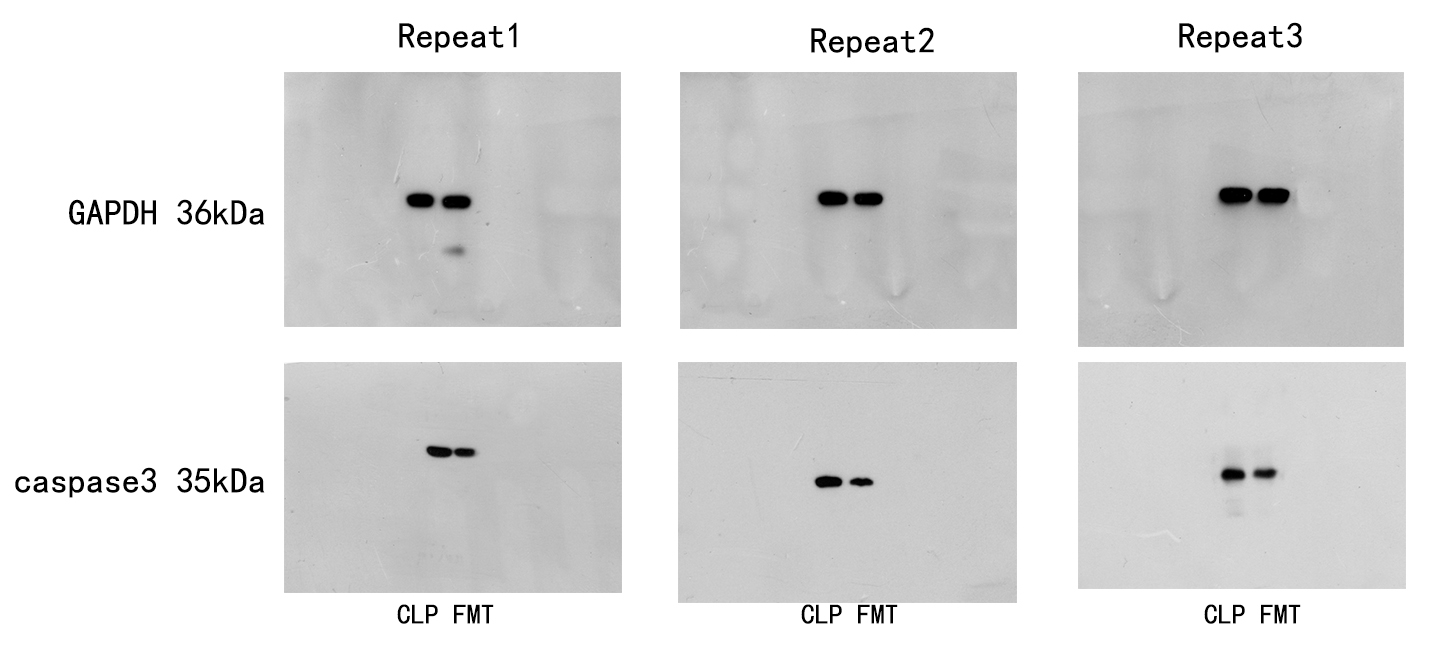


Figure 1. Relative expression of caspase 3 compared with GAPDH between the CLP group and the FMT group at 24 h.


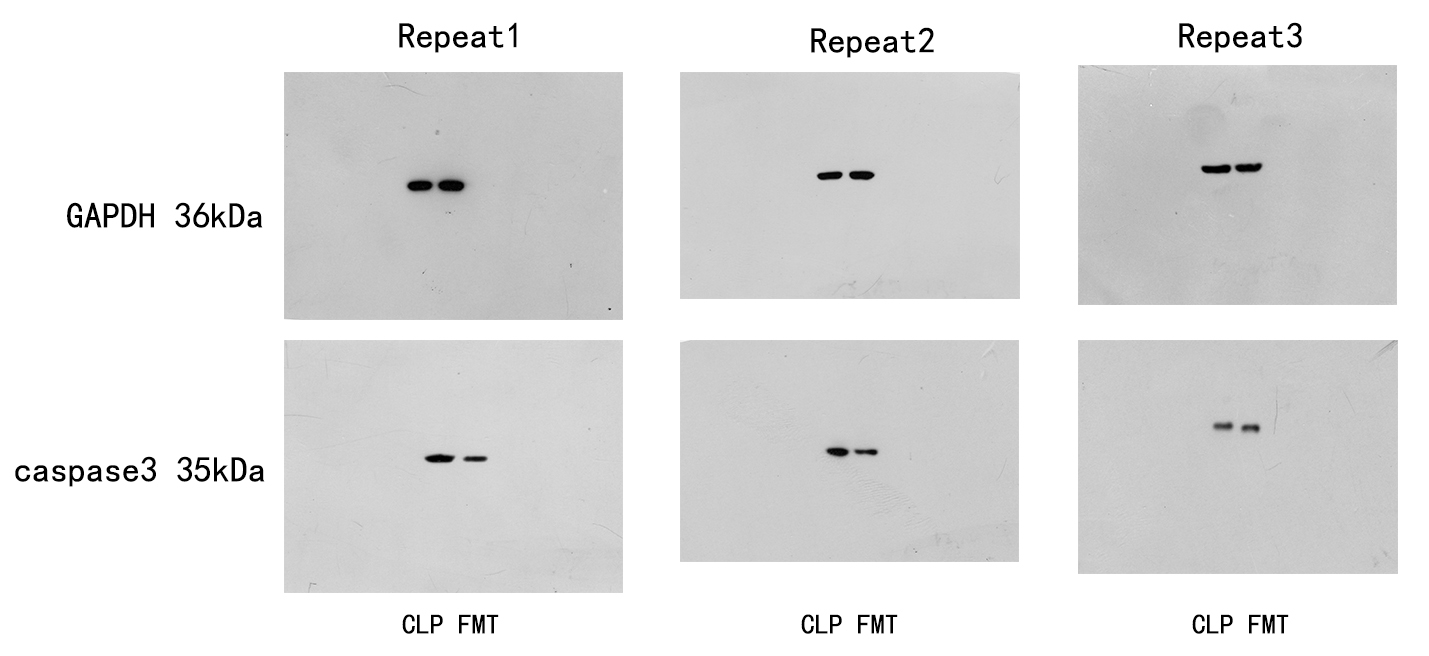


Figure 2. Relative expression of caspase 3 compared with GAPDH between the CLP group and the FMT group at 48 h.


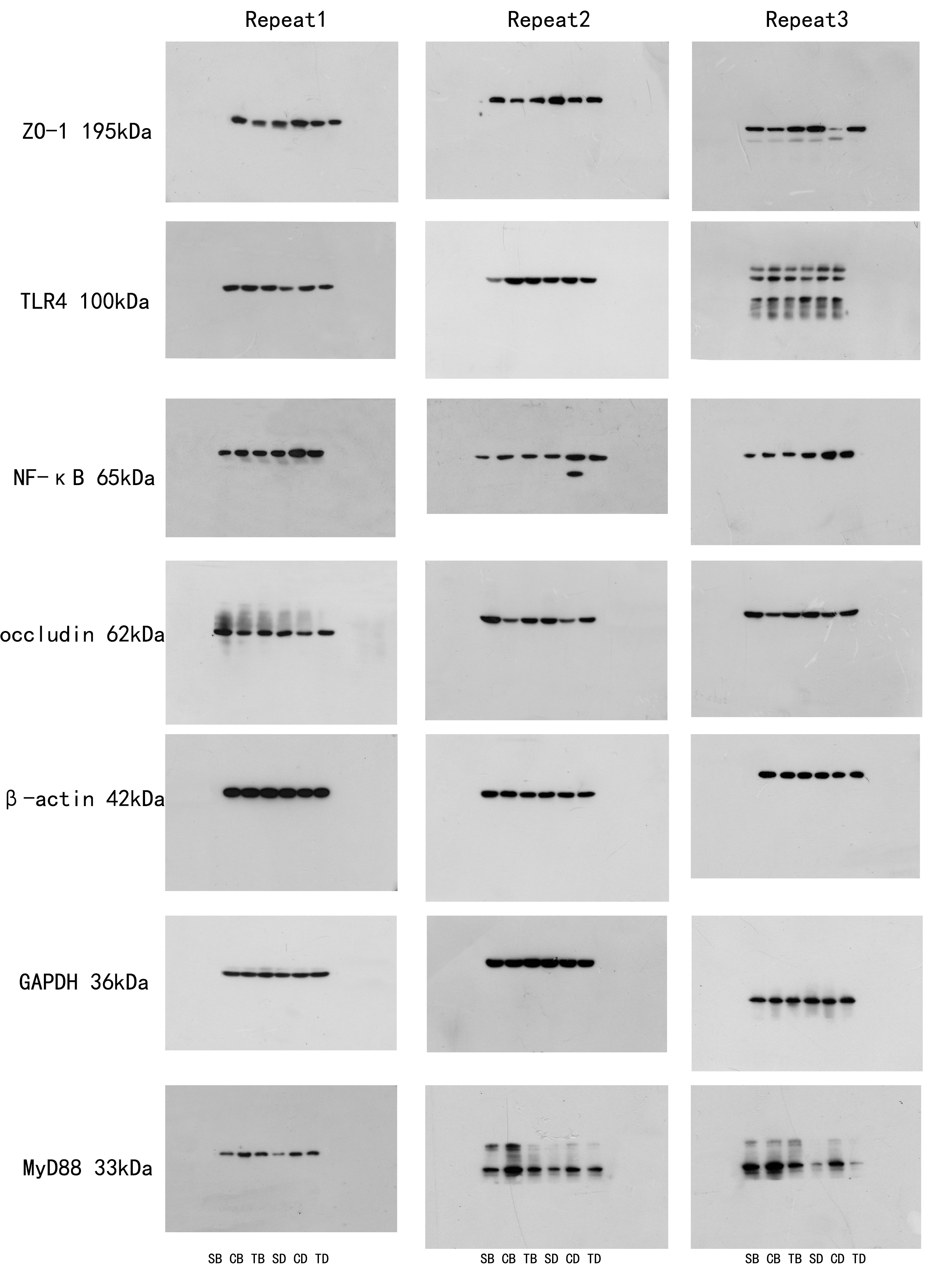


Figure 3. Relative expression of occludin and ZO-1 compared with β-actin and TLR4, MyD88, and NF-κB compared to GAPDH protein in the Sham, CLP and FMT groups at 24 or 48 h. SB, CB, TB, SD, CD, and TD represent the 24 h Sham, CLP, and FMT groups and 48 h Sham, CLP, and FMT groups, respectively.
